# A case of traumatic abdominal wall hernia with delayed bowel obstruction

**DOI:** 10.1186/s40792-015-0023-7

**Published:** 2015-02-11

**Authors:** Takeshi Nishimura, Atsunori Nakao, Ayana Okamoto, Noritomo Fujisaki, Joji Kotani

**Affiliations:** Department of Emergency, Disaster and Critical Care Medicine, Hyogo College of Medicine, Mukogawa, Nishinomiya, Hyogo 663-8501 Japan

**Keywords:** Abdominal hernia, Trauma, Ileus

## Abstract

**Background:**

Traumatic abdominal hernia is rare and difficult to diagnose from physical symptoms.

**Patient:**

A 60-year-old woman was admitted to the emergency department with complaints of vomiting after falling off a bicycle and hitting her abdomen against one of the handlebars 2 days earlier. Computed tomography (CT) demonstrated abdominal wall hernia from blunt trauma to the left upper abdomen. The patient underwent exploratory laparotomy, and the herniated bowel loop was not found to be perforated or gangrenous. Primary hernia repair without resection of the bowel loop was performed.

**Results:**

Postoperative course was uneventful.

**Conclusion:**

Surgical exploration with primary repair of the defect is the definitive treatment in the present case, as the hernia contained an incarcerated loop of small bowel. The use of abdominal CT to confirm the diagnosis before operative repair of the hernia appears to be a safe and efficacious adjunct to physical examination.

## Background

Blunt traumatic abdominal hernia is defined as a herniation through disrupted musculature and fascia without skin penetration and no evidence of a prior hernial defect at the injury site [1]. Since the skin is elastic in nature, the forces resulting from the injury lack the energy to breach the skin; however, they are sufficient to disrupt the muscle fibers and fascia, as well as the peritoneum [2]. Traumatic abdominal wall hernia is rare but quite important for experts who treat trauma patients, as a delay in diagnosis and intervention can significantly affect the outcome of patients with these hernias.

Herein, we report the case of a 60-year-old female diagnosed with traumatic abdominal wall hernia with delayed presentation of bowel obstruction, which may have been caused by low-velocity impact with a bicycle handlebar. The present case did not manifest any trauma-related symptoms apart from associated superficial skin injuries until a delayed onset of bowel obstruction. Careful follow-up using computed tomography (CT) enabled early diagnosis and treatment. Considerations in clinical and radiologic diagnosis and surgical management based on our experience are discussed.

## Case presentation

A 60-year-old woman was admitted to our hospital with complaints of vomiting and abdominal fullness following blunt abdominal trauma. She had fallen on a bicycle handle bar while bicycle riding 2 days before presentation to the emergency department. The patient developed persistent vomiting and a fever the following day. She was clinically stable with no obvious head or skeletal injuries.

Superficial contusion was present in her right iliac fossa, which bore the imprint of the bicycle handlebar. There was no edema with local rise of temperature in the overlying skin. An electrocardiogram was normal. While the abdomen appeared more distended, peritoneal signs were absent. The patient denied any previous abdominal wall defects. Her hemoglobin level remained stable, but her white blood cell count was elevated to 12,240 cells/μL. Her blood chemistry was as follows: aspartate aminotransferase, 25 U/L; alanine transferase, 20 U/L; alkaline phosphatase, 335 U/L; lactate dehydrogenase, 319 U/L; creatine kinase, 541 U/L; and C-reactive protein, 3.7 mg/dL.

**Figure 1:**
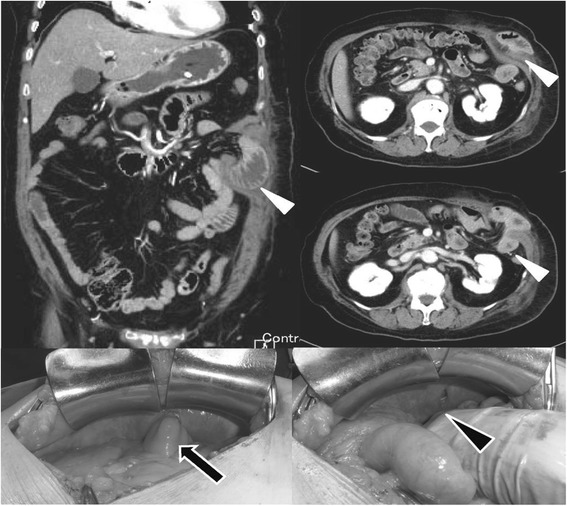
**Small bowel loops identified in the upper abdomen.** Upper panel: a CT scan of the abdomen identifying the ventral hernial defect with accompanying prolapse of the bowel (shown as white arrowhead). Bottom panel: perioperative photograph showing the impacted intestine (black arrow) and the defect in the muscle layer (black arrowhead).

Contrast-enhanced CT demonstrated an anterior abdominal wall hernia. Small bowel loops were identified within the subcutaneous tissue of the upper abdomen (Figure 1). Based on the CT scan findings, a diagnosis of incarcerated enterocele into the traumatic abdominal hernia was made and the patient underwent an emergency laparotomy. On laparotomy, blood-stained fluid was minimal. A loop of jejunum was encountered in the subcutaneous tissue of the upper abdomen. There was no covering of the peritoneal sac. The posterior rectus fascia along with the rectus muscle was transacted in a transverse fashion. Herniation through disrupted musculature and fascia with a torn peritoneum was noted. As the impacted intestine was not ischemic without protrusion, hernia repair by elective suture of the fascia and peritoneum was performed. Postoperative course was uneventful.

## Discussion

Abdominal wall disruption following blunt trauma is a rare but challenging injury in both the acute and chronic phases. Traumatic abdominal wall hernia is defined as the ‘herniation through disrupted musculature and fascia associated with adequate trauma, without skin penetration, and no evidence of a prior hernia defect at the site of injury’ [1]. The reported prevalence of traumatic abdominal hernia among trauma patients, even at dedicated trauma centers with the best facilities, is less than 1% [3]. Spencer Netto et al. retrospectively reviewed 34 patients presenting acutely with a traumatic abdominal wall hernia at a regional trauma center from January 2000 to December 2004 [3]. Their average age was 39 ± 12 years (56% male). Although no age disparity was demonstrated, the ratio of males, Injury Severity Scores, and other site injuries such as pelvic and lumbar fractures were higher compared with all blunt trauma patients. Reported mechanisms of injury have included falls, motor vehicle crashes, crush injuries, impalements, and seatbelt injuries. An analysis of recent cases indicated that most reported traumatic abdominal wall hernias resulted from seatbelt or handlebar injuries [4]. The risk of disruption of the abdominal wall is related to the size of the object, the force of impact, and the resulting distribution of the pressure load. As the skin is more elastic than the other layers of the abdominal wall, it remains intact, even though the underlying musculature and fascia are disrupted, giving rise to traumatic abdominal hernia.

Since these hernias can go undetected due to preservation of the skin overlying the hernial defect, diagnosis of traumatic abdominal wall hernia is usually made by CT scan and ultrasound to assess the injury and identify the detect in the anterior abdominal wall. CT is a useful adjunct to clinical examination and surgical exploration with primary repair as the definitive treatment in hemodynamically stable patients with traumatic abdominal wall hernia [5]. Occasionally, the mass, which is the hernia itself, is confused with a hematoma [6]. Associated injuries must be ruled out through CT. Diaphragmatic herniation is a more common complication of blunt abdominal trauma than abdominal wall herniation, although occasionally they both may co-exist [7]. Other associated injuries included pelvic fracture and rectosigmoid injuries. Once surgical treatment under general anesthesia is chosen, local exploration through an incision overlying the defect may be an option for small defects caused by low-velocity injuries. However, patients with abdominal wall hernia following high-energy trauma should undergo exploratory laparotomy through a midline incision due to the high prevalence of associated intra-abdominal injuries [8].

Whether such patients require urgent laparotomy remains controversial. Most authors advocate immediate laparotomy with repair of the defect because of the high incidence of associated intra-abdominal injury (up to 30%) and to avoid complications such as incarceration through or strangulation and subsequent morbidity. In addition, mesenteric and bowel injuries that are liable to be missed on CT scan can also be managed well in time [9]. Thus, there were no suggestions that a conservative approach be considered at any time.

On the other hand, a conservative approach would be appropriate to avoid negative outcomes in cases in which there is no associated intra-abdominal visceral injury requiring operation or bowel incarceration in the hernia [10]. We agree that immediate surgical repair is not always straightforward. As long as no associated intra-abdominal injury exists, achievement of the best surgical repair must be considered based on the size and site of the defect and the timing of repair [11]. For cases without hollow viscus injuries, relatively large defects, and tension for direct closure, primary mesh repair should be considered [12]. Spontaneous healing may occur if the defect is small and there is no other associated injury [13]. The laparoscopic approach with tension-free mesh repair of a traumatic abdominal wall hernia can be accomplished successfully using an approach similar to that taken for laparoscopic inguinal hernia repair [14]. The advantages of mesh repair are less chance of recurrence and the ability to be used in large defects where native tissue cannot be approximated. Disadvantages are that the mesh frequently causes infectious complications and may cause intestinal adhesion and erosion in the trauma setting.

Although laparotomy allows other injuries to be assessed and diagnosed at the time of operation and primary closure of defects, disadvantages include a large incision and increased postoperative recovery time. In our case, we avoided a laparoscopic approach to explore other highly suspected organ injuries in the abdominal cavity. In fact, Gutteridge et al. reviewed their cases of traumatic abdominal wall hernia and reported that none of their cases were able to be managed by laparoscopy alone [11]. The laparoscopic approach versus laparotomy must be carefully dictated by the mechanism of injury, co-existing injuries, extent of injuries, and the skill base of the surgeons at each respective center.

## Conclusions

We described a case of traumatic abdominal wall hernia requiring surgery for bowel strangulation. Patients who are diagnosed with traumatic abdominal wall hernia should be explored by CT scan as early as possible to look for intra-abdominal injuries, followed by careful examination to detect early manifestations of organ injuries. Physicians must be aware of the chances of early as well as late incarceration of the bowel in the defect leading to subsequent perforation or strangulation.

## Consent

Written informed consent was obtained from the patient for publication of this case report and any accompanying images. A copy of the written consent is available for review by the Editor-in-Chief of this journal.
